# The Transcriptional Regulator TFB-RF1 Activates Transcription of a Putative ABC Transporter in *Pyrococcus furiosus*

**DOI:** 10.3389/fmicb.2018.00838

**Published:** 2018-04-30

**Authors:** Robert Reichelt, Katharina M. A. Ruperti, Martina Kreuzer, Stefan Dexl, Michael Thomm, Winfried Hausner

**Affiliations:** Institute of Microbiology and Archaea Center, University of Regensburg, Regensburg, Germany

**Keywords:** archaea, *Pyrococcus*, transcriptional activation, TFB-RF1, ABC transporter, ChIP-seq

## Abstract

Transcription factor B recruiting factor 1 (TFB-RF1; PF1088) is a transcription regulator which activates transcription on archaeal promoters containing weak TFB recognition elements (BRE) by recruiting TFB to the promoter. The mechanism of activation is described in detail, but nothing is known about the biological function of this protein in *Pyrococcus furiosus*. The protein is located in an operon structure together with the hypothetical gene *pf1089* and western blot as well as end-point RT-PCR experiments revealed an extremely low expression rate of both proteins. Furthermore, conditions to induce the expression of the operon are not known. By introducing an additional copy of *tfb-RF1* using a *Pyrococcus* shuttle vector we could circumvent the lacking expression of both proteins under standard growth conditions as indicated by western blot as well as end-point RT-PCR experiments. A ChIP-seq experiment revealed an additional binding site of TFB-RF1 in the upstream region of the *pf1011/1012* operon, beside the expected target of the *pf1089/tfb-RF1* region. This operon codes for a putative ABC transporter which is most-related to a multidrug export system and *in vitro* analysis using gel shift assays, DNase I footprinting and *in vitro* transcription confirmed the activator function of TFB-RF1 on the corresponding promoter. These findings are also in agreement with *in vivo* data, as RT-qPCR experiments also indicate transcriptional activation of both operons. Taken together, the overexpression strategy of *tfb-RF1* enabled the identification of an additional operon of the TFB-RF1 regulon which indicates a transport-related function and provides a promising starting position to decipher the physiological function of the TFB-RF1 gene regulatory network in *P. furiosus*.

## Introduction

The core transcription machinery of Archaea resembles a simplified version of the eukaryotic RNA polymerase (RNAP) II system, which consists of only one multi-subunit RNA polymerase (RNAP). For initiation of transcription three general transcription factors (TFs), the TATA-binding protein (TBP), TFB, and TFE are necessary for recognition of the two core promoter elements, the TATA box and the TFB recognition element (BRE; Soppa, 2001; Thomm and Hausner, 2007; Grohmann and Werner, 2011; Weinzierl, 2013; Gehring et al., 2016). The first step of transcriptional initiation is the interaction of TBP with the TATA box. In the next step TFB stabilizes this complex and also interacts with the BRE upstream and with the transcription start site downstream of the TATA box (Renfrow et al., 2004). Recruitment of the RNAP completes the pre-initiation complex. The third TF, TFE, stimulates open complex formation and stabilizes early elongation complexes (Grünberg et al., 2007; Naji et al., 2007; Blombach et al., 2015). During elongation, the elongation factor Spt4/5 can displace TFE from the RNAP and stimulate processivity (Grohmann et al., 2011). Recent data indicate that Spt4/5 is recruited proximal to the transcription start site on the majority of transcription units, whereas on a subset of genes Spt4/5 is recruited later during elongation (Smollett et al., 2017).

It is interesting to note that the regulation of this eukaryotic-like transcription machinery is controlled by a system very similar to that found in Bacteria (Aravind and Koonin, 1999; Soppa, 2001; Bell, 2005; Geiduschek and Ouhammouch, 2005; Jun et al., 2011; Peeters et al., 2013; Karr, 2014). Corresponding genes are also frequently organized in operon structures and controlled by single promoters. The transcription rate of such promoters is influenced by transcription regulators, which in many cases consist of a helix-turn-helix DNA-binding motif. One general physiological function of these TFs is to sense signals from outside (like, e.g., availability of substrates or presence of toxic compounds) and translate them into a transcription dependent expression outcome. For this, these regulators often interact as dimers or oligomers with semi-palindromic DNA sequences located either upstream, within or downstream of the corresponding promoters. Bound regulators within the promoter interfere with TFB or TBP binding by steric hindrance and if bound further downstream inhibit RNAP recruitment. In both cases the regulators work as repressors and transcription is abolished on the level of initiation (Peeters et al., 2013).

In contrast, activating TFs usually bind upstream of BRE and TATA box and a detailed *in vitro* analysis of archaeal activation mechanisms revealed so far two basic principles: the activator promotes either binding of TBP or TFB (Ouhammouch et al., 2005; Ochs et al., 2012). In the first case the TATA box has strong deviations from the consensus sequence and in the second case there is an imperfect BRE. A detailed analysis of the gas vesicle regulator GvpE of *Halobacterium salinarum* revealed that this activator can even interact with both, different TBPs and TFB (Teufel and Pfeifer, 2010; Bleiholder et al., 2012).

In *Pyrococcus furiosus*, there are several TFs described in more detail: the leucine-responsive regulatory protein, Lrp, which inhibits transcription of the own gene by abrogating RNAP recruitment (Dahlke and Thomm, 2002). Phr, the regulator of the archaeal heat shock genes, has a similar mechanism (Vierke et al., 2003). In both cases, the TBP/TFB complex and the corresponding regulators can independently bind to the DNA. SurR can act as transcriptional activator and repressor controlling hydrogen and elemental sulfur metabolism (Lipscomb et al., 2009). TrmB and TrmBL1 are two sugar sensing regulators of ABC transporter genes, which are controlled by different inducers (Lee et al., 2005, 2007; Gindner et al., 2014). A detailed analysis of the *Pyrococcus* genome revealed more than 80 putative TFs, which represent about 4% of all open reading frames (Pérez-Rueda and Janga, 2010). The physiological function of almost all of these TFs and the corresponding gene regulatory networks are unknown.

One of these candidates with unknown biological function is TFB-RF1, which activates transcription by recruiting TFB to the promoter (Ochs et al., 2012). To shed light on the function of TFB-RF1, we studied the genome-wide binding sites of the regulator. We expressed the protein using a shuttle vector-based genetic system for *Pyrococcus* (Waege et al., 2010). Applying chromatin immunoprecipitation in combination with high-throughput sequencing (ChIP-seq) we identified a putative ABC transporter as an additional member of the TFB-RF1 regulatory network.

## Materials and Methods

### Strains and Growth Conditions

*Pyrococcus furiosus* was cultivated under anaerobic conditions at 85°C in ½ SME medium as described previously (Waege et al., 2010). Gelrite (1%) was added for solidification of the medium. For the comparison of the two strains, *Pfu* pYS3 containing the empty shuttle vector and *Pfu* pYS5 with *tfb-rf1* under the control of a gluconeogenic promoter, a slightly modified medium without peptone and with 0.025% yeast extract was used. Growing on starch was performed in the presence of 0.1% starch and for growing on pyruvate, the starch was replaced with 40 mM Na-pyruvate. For the selection of the shuttle vector the antibiotic simvastatin was used at a concentration of 10 μM. Each strain was cultivated with starch or with pyruvate in three independent experiments until a cell density of approximately 1 × 10^8^ cells per ml. Cell numbers were analyzed with a Thoma counting chamber (0.02 mm depth; Marienfeld, Lauda-Königshofen, Germany) using phase-contrast microscopy.

### Strains, Plasmids, and Primers

All used strains, plasmids, and primers are shown in the supplement (Supplementary Table 1).

### Construction of the Shuttle Vector pYS5 and Transformation in *P. furiosus*

Shuttle vector pYS5 was obtained according to the construction of pYS4 (Waege et al., 2010). The promoter sequence of the fructose-1,6 bisphosphatase was amplified from genomic DNA using the primers EcoRV-PF0613Pr-F and PF1088/PF0613Prom-R. For the amplification of *tfb-rf1* the primers PF1088-F and PF1088-His-R were used. The terminating region of the histone A1 gene was obtained using the primer pair His-PF1831Term-F and PF1831T-EcoRV-R. Overlapping parts of fragments were fused by single overlap extension PCR. Finally, the construct was integrated into the pYS3 vector next to the *hmgCoA* reductase gene cassette using the flanking EcoRV sites. The construction of the plasmid was verified by DNA sequencing. Transformation into *P. furiosus* cells was done according to Waege et al. (2010).

### Western Blot Analysis

Polyclonal rabbit antibodies were produced by Davids Biotechnology (Regensburg, Germany) using recombinantly expressed and purified TFB-RF1 (Ochs et al., 2012). The IgG fraction of the polyclonal antibodies was purified using an immobilized Protein G column (GE Healthcare, Little Chalfont, United Kingdom). Antibody containing fractions were pooled and dialyzed overnight against phosphate-buffered saline (PBS). For the preparation of cell extracts from the *Pyrococcus* strains, cell cultures with a volume of 20 ml and a cell density of approximately 1 × 10^8^ cells per ml were harvested, washed with 1 ml PBS and resupended in 300 μl PBS including protease inhibitor mix (cOmplete Ultra Tablets, Roche Applied Science, Mannheim, Germany). Cells lysis was induced by sonication (Sonopuls HD2070, Bandelin Electronics, Berlin, Germany), cell debris was removed by centrifugation and the protein concentrations of the supernatants were determined by Bradford assay. Western blot experiments were done as previously described (Waege et al., 2010). The signals were visualized using a Cy5-labeled secondary anti-rabbit antibody from Thermo Scientific (Waltham, MA, United States) and a fluorescence image analyzer (FLA-5000, Fuji, Japan). Each experiment was repeated three times and representative data are shown in the manuscript.

### Immunoprecipitation

ChIP experiments were carried out as described previously (Reichelt et al., 2016). *P. furiosus* cells harboring the pYS5 plasmid were cultured in a fermenter in the presence of 40 mM sodium pyruvate for formaldehyde-crosslinking. For immunoprecipitation 10 μg of purified polyclonal antibodies raised against TFB-RF1 was coupled to 50 μl Dynabeads Protein G (Thermo Fisher Scientific, Waltham, MA, United States) according to the manufacture’s instructions. After elution from the beads DNA was purified and subsequently used for library preparation. Input sample was prepared as described previously (Reichelt et al., 2016) and three replicates of immunoprecipitation (IP1, IP2, and IP3) were used for further analysis.

### Library Preparation and Sequencing

Library preparations were done according to the NEBNext^®^-ChIP-Seq library prep reagent set for Illumina protocol (New England Biolabs, Ipswich, MA, United States). For multiplex sample preparation the NEBNext^®^ Multiplex oligos (primer sets 1 and 2; New England Biolabs, Ipswich, MA, United States) were used and libraries were PCR amplified by the NEBNext^®^High Fidelity Master Mix (New England Biolabs, Ipswich, MA, United States). Libraries were pooled in equimolar ratios and sequenced using the Illumina HiSeq 2000 platform (read length = 50 b) (Illumina, San Diego, CA, United States). For further analysis only the demultiplexed and quality filtered reads were used.

### Data Processing and Peak Calling

Reads were mapped to the *P. furiosus* DSM3638 genome using Bowtie2 with default settings (Langmead et al., 2009). Aligned and unaligned reads were written to different files. The Integrative Genome Viewer (IGV) was used for visualization of short-reads genome occupancy (Robinson et al., 2011). The Bam file of aligned reads was converted to the Sam format (Li et al., 2009) and from each sample 10 million reads were randomly selected (^1^Blankenberg et al., 2010). After converting back to the Bam format samples were analyzed with the Pique software package (Wilbanks et al., 2012) using the following settings: a = 50; l = 300; ‘analysis_region’: 0 1908256 (whole genome); ‘norm_region’: 1660000 1661000 (*pfk* promoter region); and ‘mask’ 1613140 1629427 (PF1937-PF1952). Called peaks were verified by repeating the analysis using additional genomic regions or no specified genomic region as ‘norm_region.’ Downstream analyses were performed as recommended for the software. Statistical analysis was carried out using SPSS Statistics 21 (IBM). In addition, only binding sites were considered for further analysis, which were present in the three replicates of IP, which do not correspond to the ribosomal gene locus in the *P. furiosus* genome and which show higher enrichment scores than the regions corresponding to genes and promoter or terminator regions present as an additional copy on the plasmid pYS5 (Supplementary Table 2). ChIP-seq raw data are available in the ArrayExpress database^2^ under accession number E-MTAB-6618.

### RNA Isolation

*Pyrococcus furiosus* total RNA was purified using the peqGOLD TriFast^TM^ reagent (Peqlab, Erlangen, Germany). 10 to 20 ml cell culture was pelleted and cells were lysed by addition of 1 ml TriFast followed by rigorous shaking for 15 min. After adding 0.2 ml 2 M sodium acetate pH 4.0 RNA was isolated according to the manufacture’s instructions. Contaminating DNA was removed via the TURBO DNA-free^TM^ Kit (Thermo Fisher Scientific, Waltham, MA, United States). The integrity of the total RNA was assessed by formaldehyde agarose gel electrophoresis and purified RNA was stored at -80. Three independent RNA samples were prepared from both culture conditions and strains.

### Reverse Transcriptase PCR (RT-PCR) and Quantitative Real-Time PCR (RT-qPCR)

Reverse transcription was done using a QuantiTect Reverse Transcription Kit (Qiagen, Hilden, Germany) in combination with a Random Primer Mix (Promega, Madison, WI, United States). For each assay 415 ng total RNA was used. RT-PCR and RT-qPCR primer pairs were designed using the Primer3 software package (Supplementary Table 1; Untergasser et al., 2012). To confirm the absence of genomic DNA, negative control reactions containing RNA and all reagents except for the reverse transcriptase were performed (-RT cDNA).

RT-PCR reactions were assembled using +RT cDNA (1:10 diluted), -RT cDNA (1:10 diluted) or no cDNA (No Template Control; NTC) and corresponding primer pairs with a final concentration of 1 μM. Amplification was done with the Phusion DNA polymerase (New England Biolabs, Ipswich, MA, United States) and RT-PCR products were analyzed by agarose gel electrophoresis. One representative result is shown in the manuscript.

RT-qPCR reactions were assembled as triplicates in a total volume of 10 μl using the SensiMix^TM^ SYBR^®^ No-ROX Kit (Bioline, Luckenwalde, Germany). Primers were added to a final concentration of 0.3 μM and the total volume of the DNA samples in each reaction was 4 μl. The efficiency of the primer pairs, determined by serial dilutions, was in the range between 0.9 and 0.95 (Bustin et al., 2009). RT-qPCR reactions were run on a Rotorgene 6000 platform using a three step protocol: 95°C – 10′ for one cycle; 95°C – 30″, 58°C – 30″, 72°C – 30″ for 40 cycles. No template controls were used as negative controls for each primer pair and the specificity of the PCR products was verified by melt curve analysis. Data evaluation was done using the corresponding Rotorgene software package (Qiagen, Hilden, Germany). Relative expression levels are shown as log2 ratios using the reference as indicated and *pf0256* as calibrator. *Pf0256* encodes the constitutively expressed RNA polymerase subunit E′. The applicability of *pf0256* as calibrator was evaluated using various genes and *P. furiosus* wild type cells grown on pyruvate and starch (Supplementary Figure 1).

### Proteins and DNA Templates

The purification of RNAP and the TFs TBP, TFB, and TFB-RF1 was performed as described previously (Ochs et al., 2012). DNA regions of interest were obtained from genomic DNA by PCR amplification with the Phusion DNA polymerase (New England Biolabs, Ipswich, MA, United States) using corresponding primers (Supplementary Table 1). For electrophoretic mobility shift assay (EMSA) one of the primers was labeled with Cyanine-5 (Cy5) and for DNaseI footprinting one of the primers was labeled with 6-carboxyfluorescein (FAM). For *in vitro* transcription assays DNA templates were PCR amplified using unmodified primers.

### Electrophoretic Mobility Shift Assay (EMSA)

Cy5-labeled DNA and various amounts of proteins (TBP, TFB, RNAP, and TFB-RF1) were assembled in a 15 μl reaction volume according to Ochs et al. (2012). After incubation at 70°C for 15 min, protein-DNA complexes were analyzed using a non-denaturating 8% polyacrylamide gel. DNA fragments were visualized using a fluor imager (FLA-5000, Fuji, Japan). EMSAs were repeated at least two times and one representative gel is presented.

### DNase I Footprint

FAM-labeled template DNA and TFB-RF1 were incubated under the conditions used for the gel shift assay. DNase I was added for 1 to 5 min at 37°C and the reaction was stopped by the addition of 95% formamide. DNA was ethanol precipitated and resuspended in 3 μl of formamide buffer. A DNA sequencing ladder using the same primer was generated as a molecular mass standard. Samples were loaded onto a 4.5% denaturing polyacrylamide gel and analyzed using an ABI 377 DNA sequencer. DNase I footprints were repeated two times and a representative experiment is shown.

### *In Vitro* Transcription Assay

Transcription reactions were assembled in a volume of 25 μl in the presence of 10 nM template DNA, 10 nM RNAP, 238 nM TBP, 135 nM TFB, 0.25 mg/ml BSA, 440 μM ATP, 440 μM GTP, 440 μM CTP, 2.7 μM UTP, and 0.049 MBq [α-^32^P]-UTP (111 TBq/mmol) as described previously (Ochs et al., 2012). After incubation at 70°C for 10 min the RNA transcripts were purified by phenol/chloroform/isoamylalcohol extraction and separated on a denaturing 8% polyacrylamide gel. Radioactive labeled transcription products were visualized using a Phosphorimager (FLA-5000, Fuji, Japan). For quantification of the transcripts we used the AIDA Image Analyzer software (Raytest, Straubenhardt, Germany).

### Bioinformatical Analyses

The genomic sequences of 35 *Thermococcales* species were obtained from NCBI Taxonomy Browser^3^. Homologous protein searches were done by BLAST^4^. TF binding site discovery was performed using the MEME Suite (^5^Bailey et al., 2009). Predictions of transmembrane helices in proteins were done using the TMHMM Server v. 2.0^6^. Sequence logos were created by WebLogo 3 (^7^Crooks et al., 2004).

## Results

**FIGURE 1:**
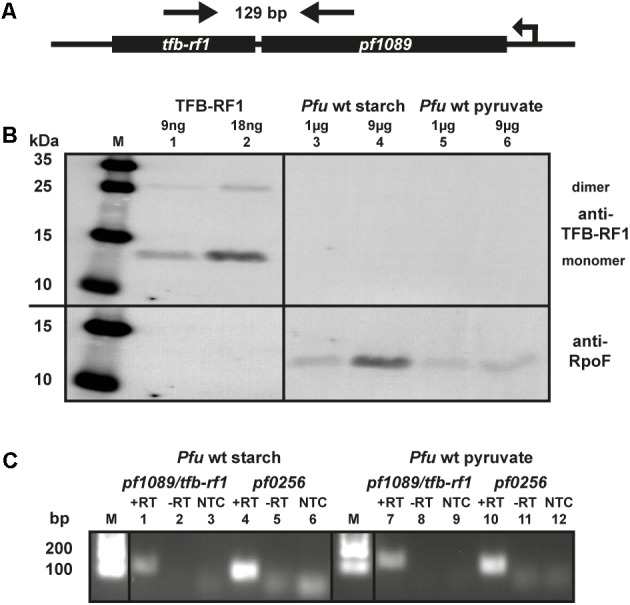
Expression of TFB-RF1 in *Pyrococcus furiosus*. **(A)** Schematic drawing of the *pf1089*/*tfb-rf1* operon. The direction of transcription and the primers used for RT-PCR are indicated by arrows. The corresponding amplicon is 129 bp and encompasses both genes. **(B)** Western blot analysis using recombinantly expressed TFB-RF1 (lanes 1 and 2) and *P. furiosus* (*Pfu*) crude extracts from cells grown on starch (lanes 3 and 4) or pyruvate (lanes 5 and 6). The molecular weight marker is shown on the left (M). Primary polyclonal antibodies raised against TFB-RF1 (upper part) and the RNA polymerase subunit F (RpoF; lower part) were used in a 1:1000 dilution. **(C)** End-point RT-PCR of the *pf1089*/*tfb-rf1* operon and *pf0256* with total RNA obtained from *P. furiosus* cells grown on starch (lanes 1 to 6) or pyruvate (lanes 7 to 12). RT-PCR was performed in the presence (+RT) or absence (–RT) of reverse transcriptase. The specificity of the PCR reaction was checked via a no template control (NTC). *Pf0256* encodes the RNAP subunit E′ and serves as housekeeping gene. The lanes with molecular weight markers are depicted with M.

The transcriptional regulator TFB-RF1 (PF1088) activates transcription on archaeal promoters containing weak BRE elements by recruiting TFB to the promoter (Ochs et al., 2012). We have described the mechanism of activation in detail, but so far we have no indication for the biological role of this protein in *P. furiosus*. The corresponding gene is located downstream in an operon structure together with the hypothetical gene *pf1089* (**Figure 1A**) and TFB-RF1 activates transcription of this operon (Ochs et al., 2012). To get an idea about the physiological function of TFB-RF1 we checked the expression of the regulator under glycolytic (starch) as well as gluconeogenic (pyruvate) conditions by western blot analysis. The TFB-RF1-specific polyclonal antibody recognized the recombinant protein (**Figure 1B**, lanes 1 and 2), but no signals were detected in crude extracts from *P. furiosus* cells grown under both conditions (**Figure 1B**, lanes 3 to 6, upper part), except control signals with an antibody raised against subunit F of the RNAP (lower part). To check the expression of the *pf1089/tfb-rf1* operon on the RNA level, end-point RT-PCR was performed. Using a PCR amplicon spanning both genes (indicated by arrows in **Figure 1A**), only a weak signal could be detected in cells grown on starch or pyruvate (**Figure 1C**, lanes 1 and 7) in comparison to the housekeeping gene *pf0256* which encodes the RNAP subunit E′ (lanes 4 and 10). These data indicate that the overall expression of the operon is extremely low and most likely special conditions are required for induction.

### Overexpression of TFB-RF1 Using the Shuttle Vector pYS5

**FIGURE 2:**
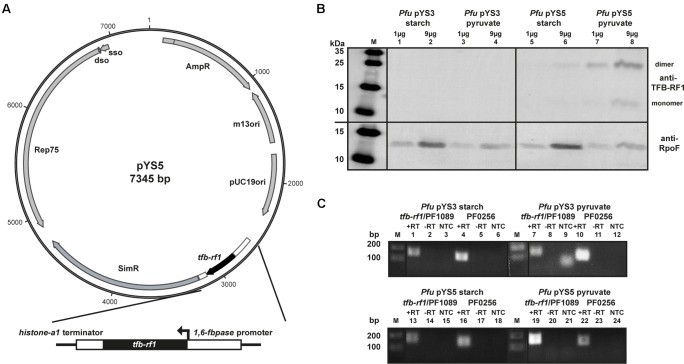
Overexpression of TFB-RF1 with the shuttle vector pYS5 in *P. furiosus*. **(A)** Schematic diagram of the shuttle vector pYS5 including the overexpression cassette for TFB-RF1 under the control of the 1,6-fructose bisphosphatase (*1,6-fbpase*) promoter and the histone A1 (*hisA1*) terminator. For the selection of the plasmid an additional copy of the *3-hydroxy-methylglutaryl coenzyme A reductase* was used to achieve resistance against the antibiotic simvastatin (Waege et al., 2010). **(B)** Western blot analysis using *P. furiosus* crude extracts from cells harboring the pYS3 plasmid grown on starch (lanes 1 and 2) or pyruvate (lanes 3 and 4) or the pYS5 plasmid grown on starch (lanes 5 and 6) or pyruvate (lanes 7 and 8). The molecular weight marker is shown on the left (M). Primary polyclonal antibodies raised against TFB-RF1 (upper part) and RpoF (lower part) were used in a 1:1000 dilution. **(C)** End-point RT-PCR of the *pf1089*/*tfb-rf1* operon and *pf0256* with total RNA obtained from *Pfu* cells harboring the pYS3 plasmid (upper panel) grown on starch (lanes 1 to 6) or pyruvate (lanes 7 to12). The lower panel contains the results of the pYS5 containing strain grown on starch (lanes 13 and 18) or pyruvate (lanes 19 and 24). Further labeling was as indicated in **Figure 1C**.

To circumvent the low expression rate of the *pf1089*/*tfb-rf1* operon we used a *Pyrococcus/Escherichia coli* shuttle vector for overexpression of TFB-RF1 (Waege et al., 2010). The constructed plasmid pYS5 contains an additional copy of *tfb-rf1* under the control of a gluconeogenic promoter and was successfully transformed into *Pyrococcus* (**Figure 2A**). A growth behavior analysis of *Pyrococcus* pYS5 under glycolytic as well as gluconeogenic conditions showed only minor differences compared to cells harboring the empty shuttle vector pYS3 as control (Supplementary Figure 2). To test for overexpression of TFB-RF1 we performed western blot experiments with both strains either grown on starch or pyruvate as described before (**Figure 2B**). Using crude extracts from *Pyrococcus* pYS3, TFB-RF1 is also not detectable similar to the wild type (**Figure 2B**, lanes 1 to 4). In contrast, using *Pyrococcus* pYS5 a weak signal could be detected in crude extract from cells grown with starch and two increased signals in crude extract from pyruvate cells (**Figure 2B**, lanes 5 to 8). The two signals correspond to the monomer and the dimer of TFB-RF1 and all attempts to achieve complete denaturation of the protein with increased SDS concentrations were not successful (data not shown). It is interesting to note that the ratio between the monomer and the dimer is the other way round in comparison to the recombinant protein produced in *E. coli* (compare **Figure 1B**, lane 2 and **Figure 2B**, lane 8).

To check if the plasmid-expressed TFB-RF1 is able to act *in trans* on the activation of the genome-located *pf1089*/*tfb-rf1* operon we performed RT-PCR reactions as described before. Using RNA from *Pyrococcus* pYS5 grown on pyruvate, an enhanced *pf1089*/*tfb-rf1* signal could be detected even stronger than the signal of the house-keeping *pf0256* (**Figure 2C**, lanes 19 and 22). As the promoter is only active under gluconeogenic conditions we obtained wild type results under glycolytic conditions (**Figure 2C**, lanes 13 and 16). This is also the case for RNA from the control strain *Pyrococcus* pYS3 (**Figure 2C**, lanes 1, 4, 7, and 10). Taken together, these results demonstrate that the strategy to bypass the missing natural expression of TFB-RF1 by introducing an additional plasmid-encoded copy of *tfb-rf1* is working and TFB-RF1 is able to stimulate transcription on corresponding promoters located in the genome.

### Genome-Wide Identification of TFB-RF1 Bindings Sites *in Vivo* by ChIP-seq

**FIGURE 3:**
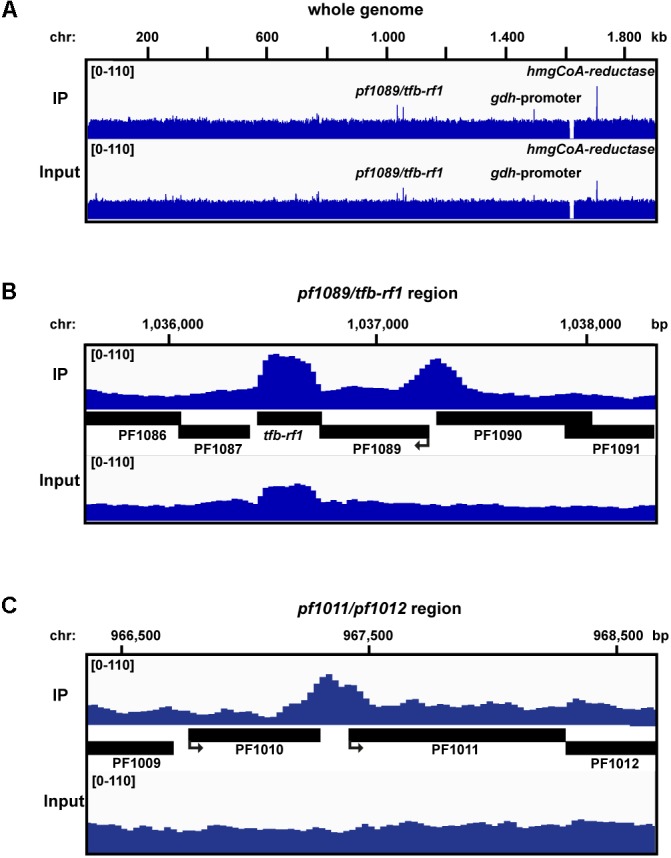
ChIP-seq analysis with *Pfu* pYS5 grown on pyruvate. Mapping of the TFB-RF1 immunoprecipitation (IP) and input sample reads was visualized using the IGV genome browser (Robinson et al., 2011) for the whole genome **(A),** the *pf1089*/*tfb-rf1* **(B),** and *pf1011/1012* **(C)** promoter regions. Prominent peaks are labeled.

To investigate if TFB-RF1 recognizes additional binding sites within the *Pyrococcus* genome we performed ChIP-seq experiments as described previously (Reichelt et al., 2016). We used formaldehyde-fixed *Pyrococcus* pYS5 cells grown on pyruvate for immunoprecipitation with the TFB-RF1-specific antibody in three replicates. The software package PIQUE was used for peak calling, which is adapted to the requirements of a peak calling algorithm suitable for binding site identification in small archaeal genomes (Supplementary Table 2; Wilbanks et al., 2012). The overview of the mapped sequences in **Figure 3A** confirmed the recently described 16 kb deletion in the *Pyrococcus* genome (Reichelt et al., 2016). Furthermore, a few prominent enriched regions were present in the immunoprecipitation as well as in the input samples. These peaks correspond to genes and promoter or terminator signals with an additional copy on the plasmid pYS5 beside the chromosome (**Figure 3A**, glutamate dehydrogenase promoter and hydroxymethlyglutaryl-CoA reductase). This leads to an enrichment of these regions in the input samples. However, two chromosomal positions were identified, which are also located in close proximity to promoter elements. The first one corresponds to the upstream region of *pf1089* and proofs that transcriptional activation of the operon (**Figure 2C**) is correlated with *in vivo* binding of TFB-RF1 upstream of *pf1089* (**Figure 3B**). The additional signal for *tfb-rf1* in both samples, IP and input DNA, resulted from overexpression on the plasmid. The second peak covers the promoter region upstream of the operon *pf1011/ pf1012*, which encode most likely an ABC transporter system (**Figure 3C**). The ChIP-seq approach indicates that the regulatory function of TFB-RF1 is not only restricted to auto-activation of its own operon, but also controls in addition operon *pf1011/ pf1012*.

### Activation of the *pf1011/pf1012* Operon by TFB-RF1 *in Vitro* and *in Vivo*

**FIGURE 4:**
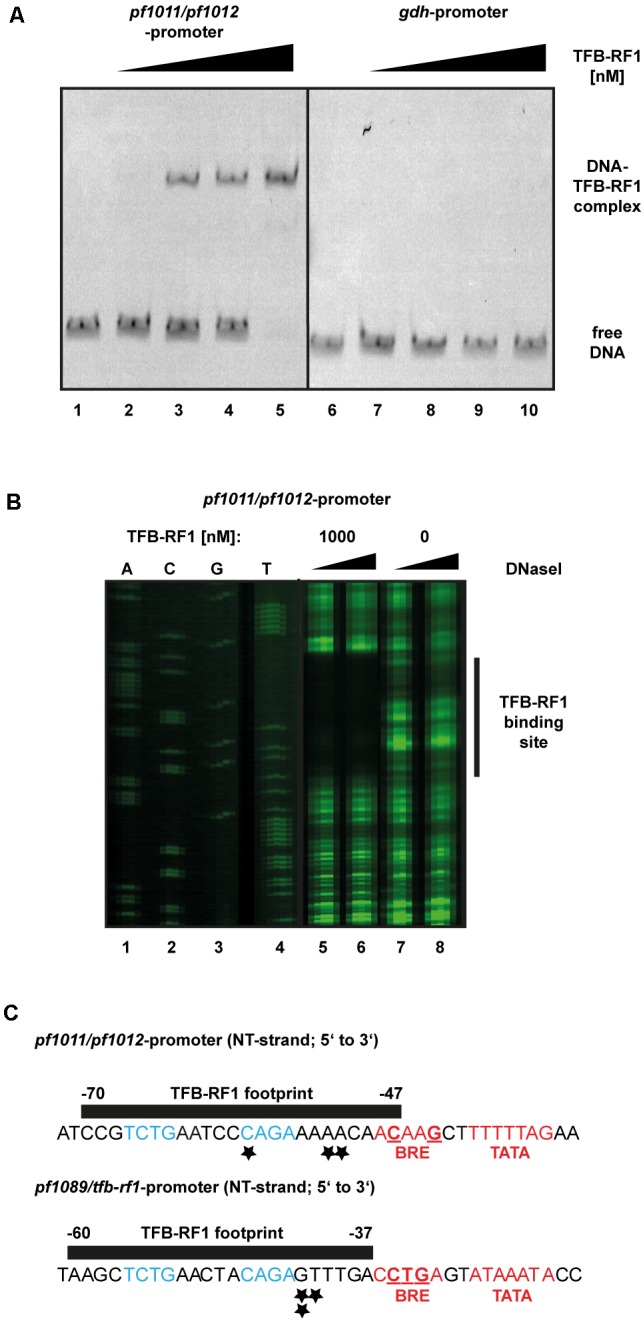
Biochemical analysis of TFB-RF1 binding in the *pf1011/1012* promoter region. **(A)** Electromobility shift assay with TFB-RF1 using the *pf1011/1012* (lanes 1 to 5) as well as the *gdh* promoter region (lanes 6 to 10) region. 11.5 nM Cy5-labeled DNA was used and the amount of TFB-RF1 was varied from 25 nM (lanes 2 and 7) to 100 nM (lanes 5 and 10) in 25 nM steps. **(B)** DNase I footprinting at the non-template strand (NT) in the *pf1011/1012* promoter region. The FAM-labeled DNA fragments (15 nM) were incubated with 0.01 (lanes 5 and 7) or 0.1 (lanes 6 and 8) units of DNase I for 5 min. The presence or absence of 1000 nM TFB-RF1 in the reaction is indicated at the top of the figure. DNA fragments were analyzed on a denaturing polyacrylamide gel using a sequencing reaction of the corresponding DNA as a size marker (lanes 1 to 4). The protected region of the DNA sequence is indicated on the left with a black bar. **(C)** Comparison of the TFB-RF1 footprint in the *pf1011/1012* promoter region (upper panel) with the known binding site of TFB-RF1 in the *pf1089*/*tfb-rf1* promoter region (lower panel). The sequences of the NT strands from 5′ to 3′ are shown and the black bars represent the protected footprint regions of TFB-RF1. The corresponding start points and end positions of the footprints are given relatively to the translation start sites. The TFB-RF1 binding sites identified via ChIP-seq are indicated by asterisks. The presumable BRE- and TATA-boxes are displayed in red and deviations from the *P. furiosus* BRE consensus sequence (van de Werken et al., 2006) are underlined. Identical nucleotides in the TFB-RF1 binding sites in both promoter regions representing the palindromic DNA binding motif are highlighted in blue.

To verify the specific interaction of TFB-RF1 with the promoter region of the newly identified operon *pf1011/ pf1012* a gel shift assay was performed (**Figure 4A**). Using the upstream region of *pf1011* as template with increasing concentrations of TFB-RF1 a specific DNA protein complex was formed (**Figure 4A**, lanes 2 to 5), but there is no interaction with the strong *gdh* promoter (lanes 7 to 10). To determine the binding site of TFB-RF1 in more detail, we also applied DNase I footprinting experiments (**Figure 4B**). TFB-RF1 protected a region on the non-template strand from -47 to -70 upstream of the translation start site (**Figure 4B**, lanes 5 to 8 and **Figure 4C**). This region shows striking sequence similarities to the one found in the *pf1089* promoter region in our previous study (**Figure 4C**, lower part; Ochs et al., 2012). Both DNA sequences contain the minimal palindromic consensus sequence 5′-TCTG-N_5_-CAGA-3′ within the footprint, which most likely serves as DNA recognition element for TFB-RF1 in both promoter regions (**Figure 4C**, blue letters). Additionally, the binding sites predicted with peak calling using PIQUE are located within both footprint regions (**Figure 4C**, black asterisks and Supplementary Table 2) which shows the high spatial resolution of the technique. The TFB-RF1 binding site in the *pf1011* promoter is located just upstream of a presumable BRE and TATA box in accordance to the *pf1089* promoter (**Figure 4C**, red letters). A comparison of the BRE of *pf1011* with the *Pyrococcus* consensus sequence 5′-VRAAA-3′ (van de Werken et al., 2006) also confirms a weak BRE, although with a reduced number of deviations compared to the consensus sequence. Therefore, we assume that *pf1011* also requires TFB-RF1 for efficient TFB recruitment and for transcriptional activation.

**FIGURE 5:**
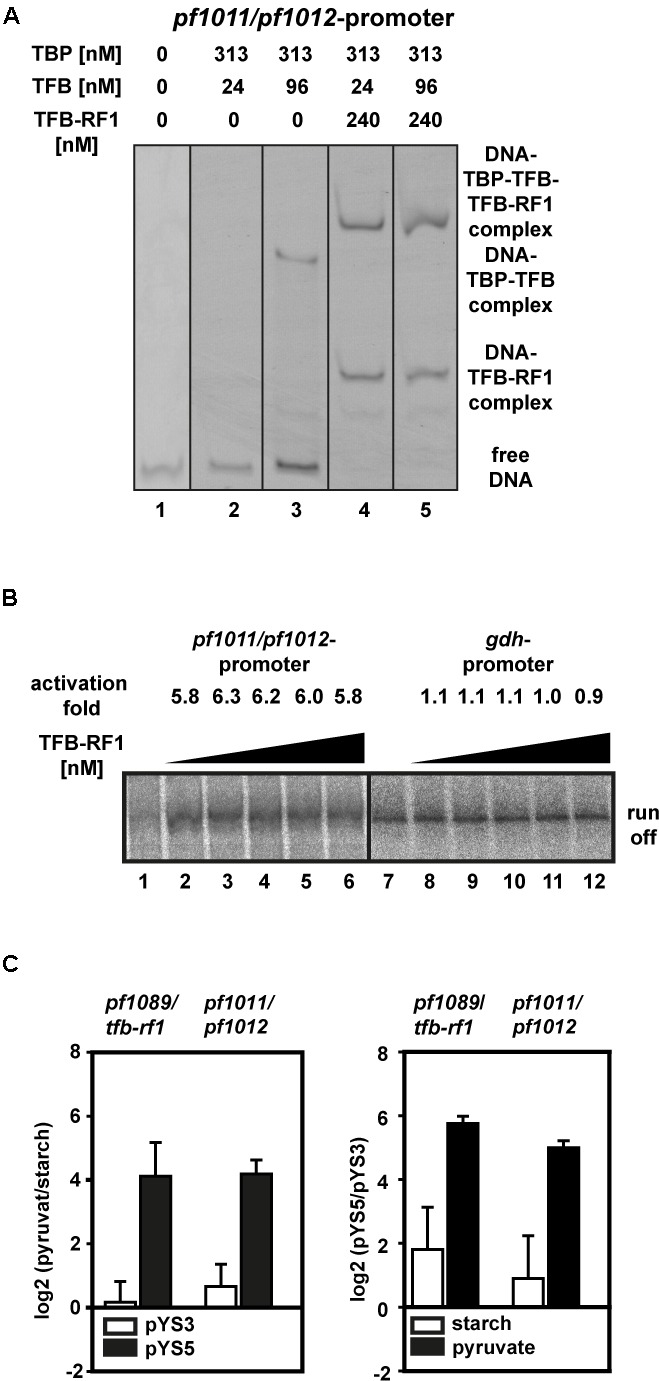
TFB-RF1-dependent activation of the *pf1011/1012* operon. **(A)** Electromobility shift assay with 11.5 nM Cy5-labeled *pf1011/1012* promoter DNA in the absence or presence of TBP, TFB, and TFB-RF1. The individual amounts are indicated on top of the figure. **(B)** Cell-free run-off transcription assays using the *pf1011/1012* (lanes 1 to 6) and the *gdh* (lanes 7 to 12) promoter as templates. The TFB-RF1 concentration was varied from 50 to 250 nM in 50 nM steps. Activation folds were calculated in comparison to run-off transcription in the absence of TFB-RF1 (lanes 1 and 7). **(C)** RT-qPCR analysis of the *pf1089*/*tfb-rf1* and *pf1011/1012* transcripts. The left panel shows the relative log2 transcript levels pyruvate versus starch with plasmid pYS3 (white bars) or pYS5 (black bars). The right panel shows the relative log2 transcript levels of the strain containing pYS5 versus pYS3 grown on starch (white bars) or pyruvate (black bars). In both cases *pf0256* was used as housekeeping gene for calibration. Standard deviations were calculated from three independent biological replicates.

To test this hypothesis we applied a gel shift assay with the upstream region of *pf1011* as DNA template. Using sufficient TBP together with a TFB concentration of 24 nM, no TBP/TFB/DNA shift was formed and even higher amounts (96 nM) did not lead to a strong shift (**Figure 5A**, lanes 2 and 3). In contrast, in the presence of TFB-RF1 a TBP/TFB/TFB-RF1/DNA complex was efficiently build even under TFB-limiting conditions (**Figure 5A**, lanes 4 and 5). Furthermore, TFB-RF1 stimulates transcription from the *pf1011* promoter in a cell-free transcription assay (**Figure 5B**, lanes 1 to 6), whereas transcription from a control promoter is not affected (**Figure 5B**, lanes 7 to 12). For the *pf1011* promoter we observed sixfold activation in comparison to sevenfold activation on the *pf1089* promoter (Ochs et al., 2012). We assume that the weak BREs shift TFB recruitment as the rate limiting step on both promoters and the increased activation in the case of the *pf1089* promoter is caused by the additional deviations of the BRE in comparison to the consensus sequence (see also **Figure 4C**). Taken together, the *in vitro* experiments clearly demonstrate that the newly identified binding site upstream of *pf1011* belongs to the TFB-RF1 regulon and also requires this protein for transcriptional activation.

To correlate our *in vitro* data with the *in vivo* situation, we isolated RNA from *Pyrococcus* pYS5 and pYS3 cells grown on starch or pyruvate and analyzed RNA levels by RT-qPCR. Both transcripts, *pf1089*/*tfb-rf1* as well as *pf1011/1012*, are highly upregulated in the pYS5 strain after growth on pyruvate (**Figure 5C**, black bars), whereas in the pYS3 strain these transcripts are almost not affected by switching from starch to pyruvate (**Figure 5C**, white bars). A direct comparison of the RNA levels between pYS5 and pYS3 strains revealed a 54-fold activation for the *pf1089*/*tfb-rf1* amplicon and 32-fold for the *pf1011/1012* amplicon under pyruvate conditions. In the presence of starch the activation is reduced to 4.5-fold for *pf1089*/*tfb-rf1* and 2.6-fold for *pf1011/1012*.

## Discussion

Research on regulatory networks of putative TFs could be very difficult, especially if nothing is known about the physiological conditions under which the regulator is active. One of such candidates is TFB-RF1 of *P. furiosus*. Although the activation mechanism is studied in detail (Ochs et al., 2012), nothing is known about the physiological role of the protein. To bypass the missing knowledge how to induce expression of the protein, we used a *Pyrococcus/E. coli* shuttle vector for overexpression of TFB-RF1 under the control of a gluconeogenic promoter (Waege et al., 2010). In combination with ChIP-seq we were able to identify the *pf1011/pf1012* operon as an additional target of the TFB-RF1 regulon. *In vitro* and *in vivo* data clearly demonstrate that the activation mechanism of TFB-RF1 on the *pf1011/pf1012* operon is also based on a weak BRE similar to the *pf1089*/*tfb-rf1* operon. A more detailed comparison of the BRE of *pf1011* with the *Pyrococcus* consensus sequence 5′-VRAAA-3′ (van de Werken et al., 2006) revealed two deviations for the *pf1011* promoter instead of three for the *pf1089* promoter. In this context it is interesting to note that the extent of TFB-RF1 induced activation correlates with the strength of the BRE. In both cases, *in vitro* and *in vivo*, the activation-fold is reduced for the *pf1011* promoter. This finding is in line with an *in vitro* mutational analysis of the BRE of the *pf1089* promoter, which indicated that a consensus BRE even abolishes TFB-RF1 induced activation (Ochs et al., 2012). Furthermore, a spontaneous mutant in the BRE of the *fla* operon in *Methanococcus maripaludis* also bypassed the need for the transcriptional activator EarA (Ding et al., 2017). Analysis of the DNA sequence from the *fla* promoter of this spontaneous mutant revealed a deletion of three adenines which converts the weak BRE in a strong one. Both organisms belong to the Euryarchaeota, but the activation of the arabinose S gene of *Sulfolobus solfataricus* (Peng et al., 2009) demonstrates that this type of activation is also present in Crenarchaeota. Overall, it seems that transcriptional activation by a TFB recruitment mechanism evolved in different archaea as well as for different genes.

To analyze in more detail, if this activation mechanism is conserved for the regulation of these two operons, we searched 35 published genome sequences of the *Thermococcales* for homologous genes.

**FIGURE 6:**
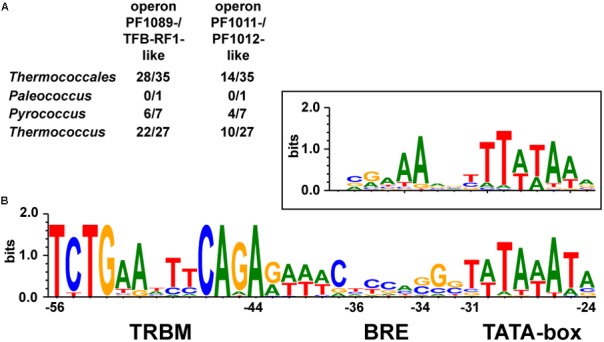
Conservation of the TFB-RF1 regulon within the *Thermococcales*. **(A)** Occurrences of the two operons encoding PF1089-/TFB-RF1-like proteins or the PF1011-/PF1012-like ABC transporter system in the genomes of 35 sequenced and annotated *Thermococci*: 1 *Palaeococcus*, 7 *Pyrococcus*, and 27 *Thermococcus* species were analyzed. **(B)** Alignment of 42 promoter regions of identified operons containing a TFB-RF1 binding motif (TRBM). The TRBM, BRE, and TATA-box regions are indicated. The box above shows the promoter consensus sequence of the BRE and TATA-box from primary transcripts of *Thermococcus kodakarensis* (Jäger et al., 2014). Positions are given relatively to the transcription start site in the *pf1089* promoter region of *P. furiosus* (Ochs et al., 2012).

We identified the *pf1089*/*tfb-rf1* operon in 28 organisms, whereas in 14 organisms both operons were present (**Figure 6A** and Supplementary Table 3). An alignment of the upstream sequences of the corresponding genes revealed three conserved elements: a bona-fide TATA box, a rather weak BRE compared to the *Thermococcus* consensus sequence (Jäger et al., 2014) and further upstream in a conserved distance the13 nt palindromic consensus sequence 5′-TCTGAANTTCAGA-3′ as TFB-RF1 Binding Motif (TRBM; **Figure 6B**). These data indicate a strong phylogenetic conservation of this gene regulatory network and for all of these promoters TFB-RF1 most likely acts as activator of transcription by supporting the recruitment of TFB to an imperfect BRE.

The identification of the *pf1011*/*pf1012* operon as an additional target of the TFB-RF1 regulon may give new implications for unraveling the physiological function. Sequence analysis revealed that the corresponding protein PF1011 contains two Pfam domains, an ATP-binding domain of ABC transporters (*ABC_tran*/PF00005) in the N-terminal region and an additional domain of unknown function (*DUF4162*/PF13732) in the C-terminal region (Finn et al., 2016). The ATPase in the N-terminal region is composed of a typical ABC transporter domain, including the Walker A and B motif, the ABC signature motif and the Q-, D-, and H-loop (Rees et al., 2009). The domain of unknown function can be mainly found at the C-terminus of bacterial or archaeal ABC transporter proteins. PF1012 is the transmembrane protein of the transporter with six predicted transmembrane segments (TMS) and belongs to the ABC2 family (*ABC2_membrane_3*/PF12698). This is a separate group of ABC transporters where the six-TMS topology resulted from intragenic duplication of a primordial three-TMS-encoding genetic element (Wang et al., 2009).

A more detailed sequence similarity search against the Transporter Classification Database (TCDB^8^) using BLAST revealed that the homologs for both proteins with the lowest E-values belong to the drug exporter-1 family (3.A.1.105; Saier et al., 2016). This family includes eukaryotic as well as prokaryotic drug transporters. The latter are mainly found in gram-positive bacteria with DrrAB and LmrA as one of the first discovered members. DrrAB is from the daunorubicin-producing strain of *Streptomyces peucetius* and LmrA is a multidrug transporter from *Lactococcus lactis* (van Veen et al., 1996; Kaur, 1997). The eukaryotic family includes the multidrug resistance P-glycoprotein, which confers resistance to anti-cancer drugs in humans (Gottesman et al., 1996), but this pump can also transport 100s of structurally unrelated hydrophobic amphipathic compounds, including therapeutic drugs, peptides and lipid-like compounds (Sharom, 2011). Due to this broad range of possible substrates, the function of the transporter in *P. furiosus* remains speculative. It is interesting to note that a computational analysis of PF1089 also revealed four TMS in its sequence, which denotes it as integral component of the membrane with unknown function. In summary, both so far identified TFB-RF1-regulated operons point to a transport-related function and provide a promising starting position to decipher the physiological function of the TFB-RF1 gene regulatory network.

## Author Contributions

MK constructed the *Pyrococcus* strains. KR did the RNA work and the western blots and SD the *in vitro* transcription experiments. RR performed the ChIP-seq experiments and the characterization of the DNA protein interactions. WH and RR wrote the manuscript. WH, RR, and MT coordinated and supervised the work. All authors agreed to the final version of the manuscript.

## Conflict of Interest Statement

The authors declare that the research was conducted in the absence of any commercial or financial relationships that could be construed as a potential conflict of interest.

## Footnotes

1 www.usegalaxy.org

2 www.ebi.ac.uk/arrayexpress

3 www.ncbi.nlm.nih.gov/Taxonomy/taxonomyhome.html/

4 blast.ncbi.nlm.nih.gov/Blast.cgi

5 meme-suite.org

6 www.cbs.dtu.dk/services/TMHMM/

7 weblogo.threeplusone.com/

8 www.tcdb.org/
