# Supplementary material for: The Transcriptional Regulator TFB-RF1 Activates Transcription of a Putative ABC Transporter in Pyrococcus furiosus
Source: Front Microbiol. 2018 Apr 30;9:838. doi: 10.3389/fmicb.2018.00838 (PMC5937170; doi:10.3389/fmicb.2018.00838)
Supplement: Supplementary file 1 [file Data_Sheet_1.pdf]

The transcriptional regulator TFB-RF1 activates transcription of a putative ABC transporter in *Pyrococcus furiosus*

Robert Reichelt, Katharina Ruperti, Martina Kreuzer, Stefan Dexl, Michael Thomm, Winfried Hausner

Institute of Microbiology and Archaea Center, University of Regensburg, Regensburg, Germany

Supplementary data

Supplementary Table 1: Used strains, plasmids and primer sequences

**P. furiosus strains**		
Designation / Alias	Description	Refernce/Source
P. furiosus wt strain DSM3638 / *Pfu* wt	*P. furiosus* wild type strain obtained from the DSMZ in 2009	(Reichelt et al., 2016)/DSMZ
P. furiosus pYS3 strain / *Pfu* pYS3	*P. furiosus* wild type strain harbouring the plasmid pYS3	(Waege et al., 2010)
P. furiosus pYS5 strain / Pfu pYS5	*P. furiosus* wild type strain harbouring the plasmid pYS5	This study
**Plasmids**		
Designation	Description	Reference/Source
pYS3	Shuttle vector containing overexpression cassette for the *hmg-CoA reductase* gene	(Waege et al., 2010)
pYS5	Shuttle vector containing overexpression cassette for the *hmg-CoA reductase* gene and inducible expression cassette for the *tfb-rf1* gene	This study
gdhC20	Amplification of control templates for EMSA and *in vitro* transcription assay	(Spitalny and Thomm, 2003)
**Primers for construction of pYS5**		
Primer	Sequence (5' to 3')	amplified gene locus
EcoRV-PF0613Pr-F	CTATTAGTATCTCCTTAACATTTCTCCAAA	promoter region *fbpase* gene
PF1088/PF0613Prom-R	CATGATCTCTTTGATTTCTTCCATTTTTTCACCTCCAGAATTTTAC	
PF1088-F	ATGGAAGAAATCAAAGAGATCATG	*tfb-rf1* gene

PF1088-His-R	TCAGTGATGGTGATGGTGATGAAAATGTATTGCATCGATTACTG	
His-PF1831Term-F	CATCACCATCACCATCACTGAAATCTTTTTAGCACTT	termination region histone A1 gene
PF1831T-EcoRV-R	TCAATTGATATCACCCTAGAAAAAGATAAGC	
**Primers for RT-PCR and RT-qPCR**		
Primer	Sequence (5' to 3')	amplified gene locus
PF0255FW	TCCACTCGAATTTTCCAAGG	Pf0256 gene encoding the RNA polymerase subunit E'
PF0255RW	GCGATTTGGTGAGAGCTAGG	
PF108889RTFW	TCCCAGGGTGTGAGATTTCA	operon PF1089/PF1088 encoding a TMS-containing protein and TFB-RF1
PF108889RTRW	CACTTGGGAACTTGCGACAG	
PF101112RTFW	AAATACCACCATCTTTTCACGC	operon PF1011/PF1012 encoding a ABC transporter system
PF101112RTRW	GGAGCCAACACTTGAAGATGT	
PF1874FW	AAGCTGCCCTTGGAAAAGAT	gene PF1874 encoding the glyceraldehyde-3-phosphate dehydrogenase
PF1874RW	ATCGGCAGCTCTCCTAATCA	
PF1784FW	CACCATCAACCATTTCACCA	gene PF1784 encoding the phosphofructokinase
PF1784RW	CCCCATTCGGGAAGATTTAT	
Rrna16s4f	GGCGACGGTAGGTCAGTATG	16S rRNA gene
Rrna16s4r	ATTGTCCCGCCCATTGTAG	
PF0983FW	ATTTGCATGGGCATTTCATT	gene PF0983 encoding sliding clamp
PF0983RW	GGACATCGAGGTTCAAGAGG	
**Primers for amplification of DNA templates**		
Primer	Sequence (5' to 3')	amplified gene locus
pf1011bF	TAT AGC TAC CAA TAT TCC TTT C	promoter region gene PF1011
pf1011bR	ACT ATT TTG GAA ATT AGT TAA AGC	
For EMSA the forward (F) primer was labeled with Cyanine-5 (Cy5) and for DNaseI footprinting the forward (F) primer was labeled with 6-carboxyfluorescein (FAM)		
PF1011-RW	AGT GTT GTT TTT CCA GCC CC	

Supplementary Table 2:

Immunoprecipitation 1 (IP1)				Immunoprecipitation 2 (IP2)				Immunoprecipitation 3 (IP3)			
start	stop	binds_at	enrichment_ratio	start	stop	binds_at	enrichment_ratio	start	stop	binds_at	enrichment_ratio
137327	138043	137685	1,8268821	141029	141689	141359	1,57859221	1036937	1037626	1037281	1,60994618
140504	141145	140824	1,8147964	137395	138069	137732	1,57145231	967017	967705	967361	1,36681206
138702	139406	139054	1,77774936	1036938	1037622	1037280	1,55609991	1494628	1495313	1494970	1,34664116
137019	137720	137369	1,72856377	140490	141176	140833	1,54972107	1705466	1706157	1705811	1,33751615
139841	140528	140184	1,70390226	138768	139442	139105	1,46696779	1036566	1037256	1036911	1,3170127
138947	139634	139290	1,70351371	138952	139642	139297	1,42431926	1494835	1495492	1495163	1,28806734
138407	139053	138730	1,68504366	139851	140543	140197	1,41340998	1705024	1705691	1705357	1,28230222
138110	138800	138455	1,65766942	967011	967698	967354	1,36964947	141023	141714	141368	1,261607
136803	137435	137119	1,64800627	136696	137342	137019	1,29146637	354272	354974	354623	1,21373203
139492	140181	139836	1,64365501	1705286	1706003	1705644	1,28150964	1494348	1494968	1494658	1,19876535
1036934	1037629	1037281	1,57620193	141393	142092	141742	1,27699453	138729	139411	139070	1,19685705
1036558	1037273	1036915	1,36626926	1705012	1705710	1705361	1,24447842	1781776	1782474	1782125	1,19620931
967018	967703	967360	1,3521136	1705579	1706180	1705879	1,24379643	199276	199891	199583	1,19522879
1705442	1706091	1705766	1,31374626	296955	297642	297298	1,24124874	61120	61829	61474	1,19417627
1494418	1495028	1494723	1,29081756	1494624	1495312	1494968	1,16243187	885170	885831	885500	1,19391079
1494628	1495311	1494969	1,28790788	1115239	1115886	1115562	1,16191946	525651	526321	525986	1,19244555
296954	297658	297306	1,27880631	1574433	1575099	1574766	1,15032725	1796980	1797637	1797308	1,1915117
1494836	1495511	1495173	1,2360924	1574195	1574860	1574527	1,14534329	185904	186594	186249	1,19086105
1036217	1036856	1036536	1,23330234	1360139	1360838	1360488	1,14411166	1843373	1844051	1843712	1,1830323
525805	526517	526161	1,20930402	580390	581049	580719	1,14173612	296974	297675	297324	1,17483167
61709	62404	62056	1,20010864	1347467	1348140	1347803	1,13578902	1736286	1736983	1736634	1,17401198
710404	711070	710737	1,19423209	737799	738491	738145	1,1350539	737820	738504	738162	1,17215176
1360316	1360990	1360653	1,19096634	314893	315553	315223	1,13158184	372981	373695	373338	1,17187688
324839	325547	325193	1,18809209	354288	354968	354628	1,12934178	584903	585569	585236	1,17034473
1557616	1558269	1557942	1,18410587	324832	325541	325186	1,12664724	1823315	1823970	1823642	1,16939222

1704588	1705289	1704938	1,18391667	1864689	1865321	1865005	1,12299986	1843161	1843863	1843512	1,16678388
1823315	1824013	1823664	1,18062101	714366	715067	714716	1,1189637	505475	506150	505812	1,16678168
315078	315785	315431	1,17065636	897251	897955	897603	1,11482183	1796703	1797386	1797044	1,16495071
1070612	1071309	1070960	1,16946329	721461	722068	721764	1,11378849	138507	139175	138841	1,16415952
737815	738499	738157	1,16761473	505472	506153	505812	1,11067455	879517	880201	879859	1,16110448
729793	730480	730136	1,1657835	897526	898230	897878	1,10850765	346794	347484	347139	1,16082145
384394	385067	384730	1,16423017	373002	373697	373349	1,10819678	1431649	1432339	1431994	1,15992553
61116	61789	61452	1,16103245	199286	199897	199591	1,10501578	1830613	1831255	1830934	1,15988711
937069	937736	937402	1,16103114	11567	12282	11924	1,10407688	714346	715038	714692	1,15852709
1148815	1149490	1149152	1,15791649	1851862	1852545	1852203	1,10403074	1115201	1115916	1115558	1,15808676
765435	766070	765752	1,15650417	1494328	1494937	1494632	1,10246471	738138	738804	738471	1,15792385
1800501	1801185	1800843	1,15274546	732128	732780	732454	1,10181007	1574398	1575082	1574740	1,15261723
384045	384749	384397	1,15021101	1192789	1193474	1193131	1,10141628	1851870	1852549	1852209	1,15075506
64100	64798	64449	1,14788441	750212	750904	750558	1,10096112	440547	441202	440874	1,14936373
212251	212924	212587	1,14567482	136396	137069	136732	1,10048817	817207	817883	817545	1,14812286

ChIP enriched regions of the three replicates of immunoprecipitation (IP1, IP2 and IP3) using antibodies against TFB-RF1. The chromosomal start and stop positions of each enriched region are shown and the presumable transcription factor binding site in the center of the peak is given (binds_at). The binding sites were sorted according to their enrichment ratios and the table includes the top 40 of each experiment. The enriched regions presented in all three replicates with the highest scores are the upstream region of the genes PF1089 (Chromosome: 1036934 – 1037622) and PF1011 (Chromosome: 967011 – 967703) (highlighted in green). The subsequently enriched regions correspond to genes and promoter or terminator regions present as an additional copy on the plasmid pYS5 (highlighted in yellow). As these regions have no binding site for TFB-RF1, we have used these enrichment ratios as cut-off for specifically TFB-RF1-ChIP enriched regions. To ensure that this manually applied cut-off did not exclude additional TFB-RF1 binding sites beneath this cut-off a FIMO search was done using the TRBM and the enriched regions of each IP (data not shown). This analysis confirmed the unique presence of the TRMB in the two upstream regions of the genes PF1089 and PF1011.

Supplementary Table 3: Sequence analysis of the TFB-RF1 regulatory network within the *Thermococcales.*

	Genus	TFB-RF1 (BLAST)	PF1089 (TMHMM)	TRBM (5'- 3')	PF1011 (BLAST)	PF1012 (BLAST)	TRBM (5'- 3')
1	*Palaeococcuspacificus*	-	-	-	-	-	-
2	*PyrococcusabyssiGE5*	PAB_RS04935	PAB_RS04930	tttgcgttacaaa	PAB_RS03525	PAB_RS03520	tctggattccata
3	*PyrococcusfuriosusDSM3638*	PF_RS05455	PF_RS05460	tctgaactacaga	PF_RS05080	PF_RS05085	tctgaatcccaga
4	*PyrococcushorikoshiiOT3*	PH_RS04980	PH_RS04985	tctgaattgcaga	-	-	-
5	*Pyrococcuskukulkanii*	TQ32_RS05675	TQ32_RS05670	tctgtaaaccaga	TQ32_RS07165	TQ32_RS07170	tctgtattccaga
6	*PyrococcusyayanosiiCH1*	PYCH_RS01655	PYCH_RS01650	tctgagcttcaga	-	-	-
7	*Pyrococcussp.NA2*	PNA2_RS07395	PNA2_RS07400	tctgaatttcaga	PNA2_RS04965	PNA2_RS04960	tttgaaattcaaa
8	*Pyrococcussp.ST04*	-	-	-	-	-	-
9	*ThermococcusbarophilusMP*	TERMP_RS09495	TERMP_RS09500	tctgaatttcaga	-	-	-
10	*Thermococcusbarossii*	A3L01_RS09360	A3L01_RS09365	tctgaagttcaga	A3L01_RS02065	A3L01_RS02060	tttgaaattcaaa
11	*Thermococcusceler*	A3L02_RS07210	A3L02_RS07205	tctgtatttcaga	-	-	-
12	*Thermococcuschitonophagus*	CHITON_RS03715	CHITON_RS03720	tctgtagaccaga	CHITON_RS00310	CHITON_RS00305	tctgtactccaga
13	*Thermococcuscleftensis*	CL1_RS05205	CL1_RS05200	tctgaagttcaga	-	-	-
14	*Thermococcuseurythermalis*	TEU_RS02670	TEU_RS02675	tctgaaattcaga	-	-	-
15	*ThermococcusgammatoleransEJ3*	TGAM_RS00140	TGAM_RS00135	tctgagattcaga	TGAM_RS08595	TGAM_RS08590	tctgaactccaga
16	*Thermococcusgorgonarius*	A3K92_RS09230	A3K92_RS09235	tctgaatttcaga	-	-	-
17	*ThermococcusguaymasensisDSM11113*	X802_RS04045	X802_RS04040	tctgaaattcaga	-	-	-
18	*ThermococcuskodakarensisKOD1*	TK_RS09140	TK_RS09135	tctgaaattcaga	-	-	-
19	*ThermococcuslitoralisDSM5473*	OCC_RS00190	OCC_RS00195	tctgaaattcaga	OCC_RS05450	OCC_RS05445	tctgaacttcaga
20	*Thermococcusnautili*	BD01_RS06330	BD01_RS06335	tttgcgccccaaa	-	-	-
21	*ThermococcusonnurineusNA1*	-	-	-	-	-	-
22	*Thermococcuspacificus*	A3L08_RS02280	A3L08_RS02285	tctgaagttcaga	-	-	-
23	*Thermococcusparalvinellae*	TES1_RS09800	TES1_RS09805	tctgaatttcaga	-	-	-
24	*Thermococcuspeptonophilus*	A0127_RS06040	A0127_RS06045	tctgaaattcaga	-	-	-

25	*Thermococcuspiezophilus*	-	-	-	-	-	-
26	*Thermococcusprofundus*	A3L09_RS01760	A3L09_RS01765	tctgaaatccaga	A3L09_RS03785	A3L09_RS03780	tctgaaccacaga
27	*Thermococcusradiotolerans*	A3L10_RS08810	A3L10_RS08815	tctgaaactcaga	A3L10_RS04445	A3L10_RS04450	tctgaatctcaga
28	*ThermococcussibiricusMM739*	-	-	-	-	-	-
29	*Thermococcussiculi*	A3L11_RS02845	A3L11_RS02850	tctgaaattcaga	A3L11_RS05905	A3L11_RS05900	tctgaatttcaga
30	*Thermococcusthioreducens*	A3L14_RS00390	A3L14_RS00395	tctgggattcaga	-	-	-
31	*Thermococcussp.2319x1*	-	-	-	-	-	
32	*Thermococcussp.4557*	GQS_06870	GQS_06865	tctgaaattcaga	GQS_03830	GQS_03835	tctgaatctcaga
33	*Thermococcussp.5-4*	CDI07_09280	CDI07_09285	tctgaaactcaga	CDI07_02050	CDI07_02045	tctgaatctcaga
34	*Thermococcussp.AM4*	TAM4_1170	TAM4_2435	tctgaaattcaga	TAM4_1798	TAM4_1654	tctgaattccaga
35	*Thermococcussp.P6*	-	-	-	-	-	-

Sequence analysis of the TFB-RF1 regulatory network within the *Thermococcales*. The genomic sequences of 35 *Thermococcales* species were obtained using the NCBI Taxonomy Browser (www.ncbi.nlm.nih.gov/Taxonomy/taxonomyhome.html/). Homologous proteins of TFB-RF1 within these genomes were identified by BLAST search (blast.ncbi.nlm.nih.gov/Blast.cgi). It is interesting to note that the sequence conserveration of all co-transcribed genes similar to *pf1089* in *P. furiosus* is limited to the presence of four to six trans-membrane helices. The regions 200 bp upstream of the start codon of these presumably co-transcribed genes were used for transcription factor binding site search using MEME Suite (meme-suite.org; Bailey et al., 2009). This analysis identified a binding motif with the consensus sequence 5'-TCTGAAHTYCAGA-3' in all 28 sites designated as TFB-RF1 Binding Motif (TRBM). Moreover, homologous ABC-transporter systems according to the genes *pf1011* and *pf1012* were identified by BLAST in 14 of the 28 TFB-RF1 containing *Thermococcales* genomes. Finally, all 42 identified promoter regions were aligned based on the TRBM and visualized using WebLogo 3 (Fig. 6; weblogo.threeplusone.com/; Crooks et al., 2004).

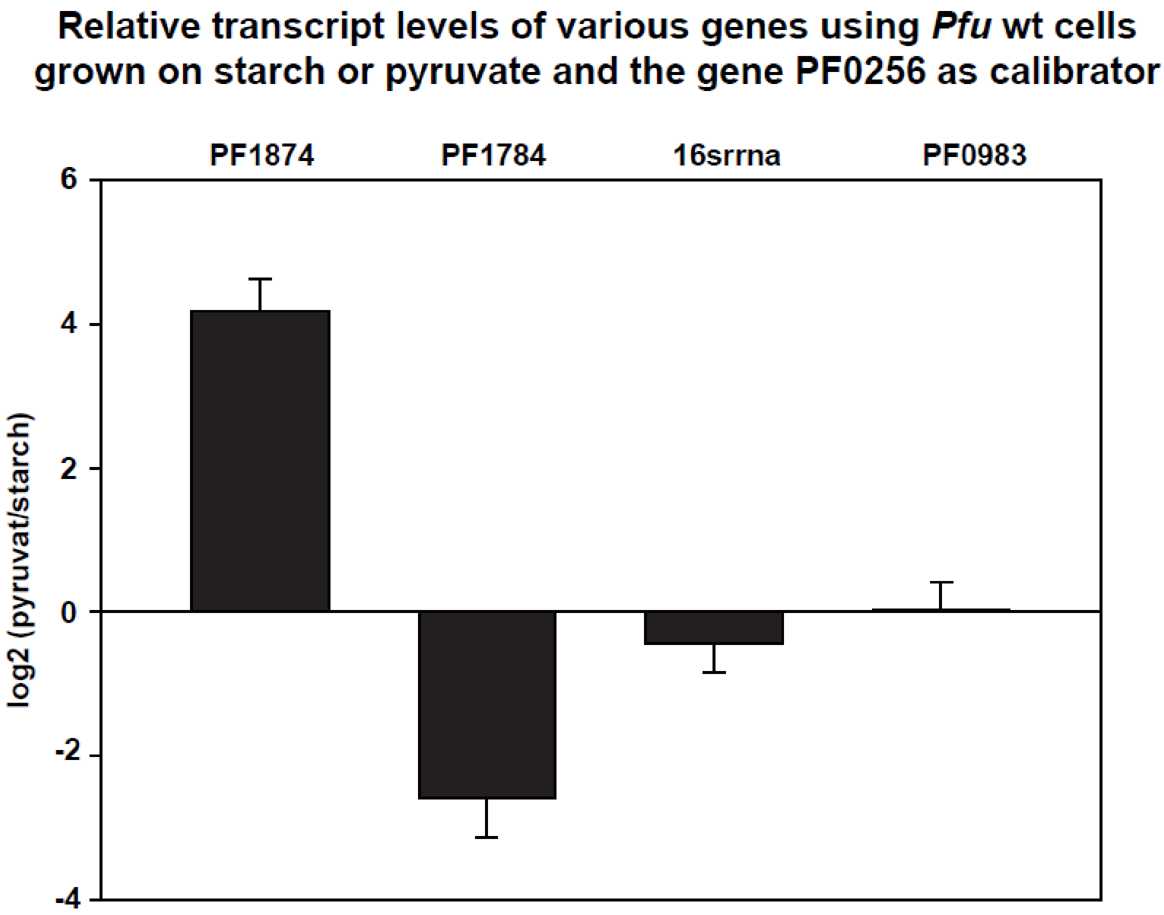

Supplementary Figure 1: Evaluation of the applicability of gene *pf0256* encoding the RNA polymerase subunit E' as housekeeper for calibration in RT-qPCR experiments. The log2 ratios of various genes are shown comparing relative RNA levels from *P. furiosus* wt cells grown on pyruvate versus starch and PF0256 as calibrator. As expected, the transcript levels of the gene *pf1874* encoding the gluconeogenic enzyme glyceraldehyde-3-phosphate dehydrogenase are upregulated after growth on pyruvate, whereas the transcript levels of the gene *pf1874* encoding the mainly glycolytic enzyme phosphofructokinase are downregulated after growth on pyruvate (Schut et al., 2003). In contrast, the transcript levels of the *16S rrna* gene are only slightly affected by growth under the two conditions. Finally, the transcript levels of the gene *pf0983* encoding the sliding clamp subunit of the DNA polymerase, which was already used by others as housekeeping gene for *P. furiosus* (Lipscomb et al., 2009), is completely unaffected by growth under the two conditions. These results clearly proof the applicability of gene *pf0256* as housekeeper for calibration in RT-qPCR experiments. The standard deviations are calculated from three independent biological replicates.

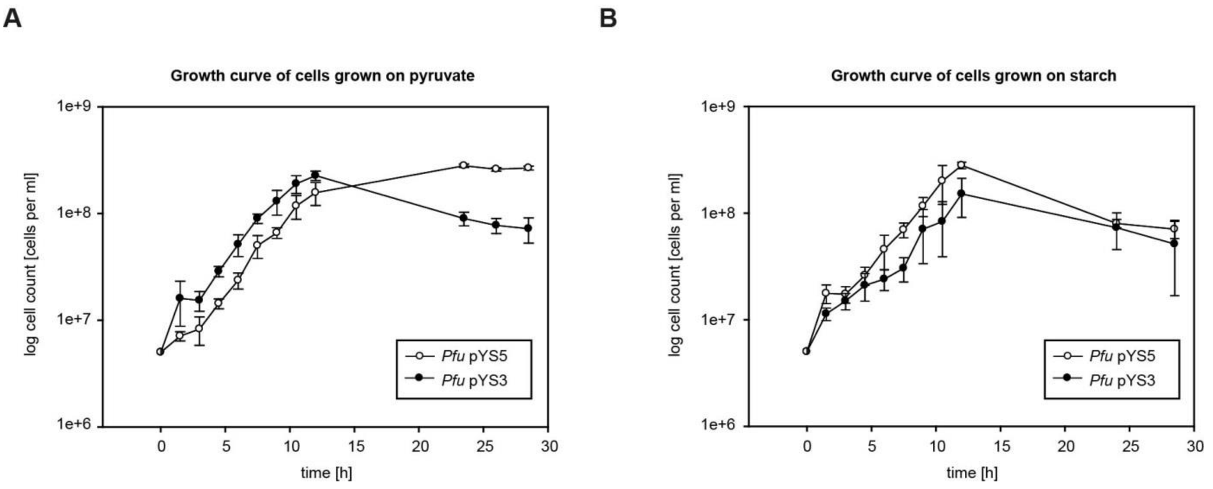

Supplementary Figure 2: Growth analysis of *Pyrococcus* strains on pyruvate (**A**) or starch (**B**). Each growth experiment was performed in triplicates, cell numbers were analyzed with a Thoma counting chamber. The results were shown in a diagram with the corresponding standard deviations.

References:

Bailey, T. L., Boden, M., Buske, F. A., Frith, M., Grant, C. E., Clementi, L., et al. (2009). MEME SUITE: tools for motif discovery and searching. *Nucleic acids research* 37, 8. doi: 10.1093/nar/gkp335

Crooks, G. E., Hon, G., Chandonia, J.-M., and Brenner, S. E. (2004). WebLogo: a sequence logo generator. *Genome research* 14, 1188–1190. doi: 10.1101/gr.849004

Lipscomb, G. L., Keese, A. M., Cowart, D. M., Schut, G. J., Thomm, M., Adams, M. W. W., et al. (2009). SurR: a transcriptional activator and repressor controlling hydrogen and elemental sulphur metabolism in Pyrococcus furiosus. *Molecular microbiology* 71, 332–349. doi: 10.1111/j.1365-2958.2008.06525.x

Reichelt, R., Gindner, A., Thomm, M., and Hausner, W. (2016). Genome-wide binding analysis of the transcriptional regulator TrmBL1 in Pyrococcus furiosus. *BMC genomics* 17, 40. doi: 10.1186/s12864-015-2360-0

Schut, G. J., Brehm, S. D., Datta, S., and Adams, M. W. W. (2003). Whole-genome DNA microarray analysis of a hyperthermophile and an archaeon: Pyrococcus furiosus grown on carbohydrates or peptides. *Journal of bacteriology* 185, 3935–3947.

Spitalny, P., and Thomm, M. (2003). Analysis of the open region and of DNA-protein contacts of archaeal RNA polymerase transcription complexes during transition from initiation to elongation. *The Journal of biological chemistry* 278, 30497–30505. doi: 10.1074/jbc.M303633200

Waege, I., Schmid, G., Thumann, S., Thomm, M., and Hausner, W. (2010). Shuttle vector-based transformation system for Pyrococcus furiosus. *Applied and environmental microbiology* 76, 3308–3313. doi: 10.1128/AEM.01951-09
